# Spatiotemporal based response for methylene blue removal using surface modified calcium carbonate microspheres coated with *Bacillus* sp.†

**DOI:** 10.1039/d2ra05466c

**Published:** 2023-01-10

**Authors:** Noha M. Deghiedy, Hanan S. El-Bastawisy, Ola M. Gomaa

**Affiliations:** a Radiation Polymer Department, National Center for Radiation Research and Technology (NCRRT), Egyptian Atomic Energy Authority (EAEA) Cairo Egypt; b Drug Radiation Research Department, National Center for Radiation Research and Technology (NCRRT), Egyptian Atomic Energy Authority (EAEA) Cairo Egypt; c Radiation Microbiology Department, National Center for Radiation Research and Technology (NCRRT), Egyptian Atomic Energy Authority (EAEA) Cairo Egypt ola_gomaa@hotmail.com

## Abstract

Calcium carbonate microspheres are attractive for their biocompatibility, high loading capacity and easy preparation. They can be used in biomedicine and catalytic applications. In the present work, calcium carbonate microspheres were surface modified with polyvinylpyrrolidone (PVP) followed by irradiation at 5 kGy prior to coating with *Bacillus* sp. cells. To provide cell protection and internal energy storage, polyhydroxybutyrate (PHB) was induced using 3 factors 2 levels factorial design where the order of effect on PHB% was pH > incubation time > glucose concentration. The highest production was 81.68 PHB% at pH 9, 20 g L^−1^ glucose and 4 days incubation time. *Bacillus* sp. cells grown under PHB optimal conditions were used to coat the surface modified calcium carbonate microspheres. Characterization was performed using X-ray diffraction, Fourier Transform Infrared Spectroscopy, Dynamic light Scattering, Zeta potential and Scanning Electron Microscopy. The results obtained confirm the formation and coating of microspheres of 2.34 μm and −16 mV. The prepared microspheres were used in bioremoval of methylene blue dye, the results showed spatiotemporal response for MB-microsphere interaction, where PHB induced *Bacillus* sp. coated microspheres initially adsorb MB to its outer surface within 1 h but decolorization takes place when the incubation time extends to 18 h. The microspheres can be reused up to 3 times with the same efficiency and with no desorption. These results suggest that the surface modified calcium carbonate can be tailored according to the requirement which can be delivery of biomaterial, bioadsorption or bioremediation.

## Abstract 2

Calcium carbonate microspheres are attractive for their biocompatibility, high loading capacity and easy preparation.

## Graphical abstract

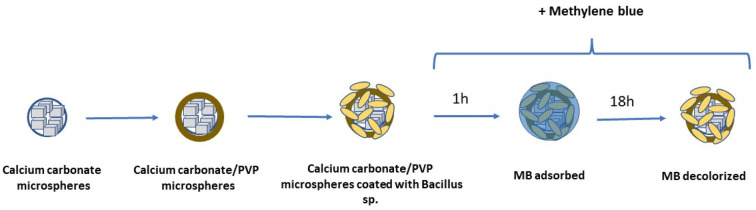

## Introduction

Methylene blue is widely used in dyeing textiles such as linen and silk and its release has been known to cause adverse health effects that include nausea, diarrhea, difficulty in breathing and eye burns.^1^ Methylene blue was also reported for its antiseptic properties and therefore is used to treat malaria, septic shock, methheamoglobinaemia and treatment of urinary tract infection and recently proposed for inhibiting cytokines produced during COVID19 infection.^2^ Its use showed significant improvement in respiratory activity that is usually compromised during COVID19 infection.^3^ There are different ways to remove methylene blue from aqueous media, these methods include removal using advanced oxidative processes, filtration, and reverse osmosis but in recent years, adsorption using different material has proven very effective in dye removal since the use of adsorptive material is usually effective, reusable and in many cases cheap. Adsorptive material such as nanocomposite,^4^ coffee waste,^5^ alginate hydrogel beads substantiated by graphene oxide,^6^ banana stem based activated carbon,^7^ natural clay^8^ are among the examples of adsorbent material that are effective in dye removal. But one of the interesting materials that can be successfully used in dye removal is calcium carbonate (CaCO_3_) microspheres.^9^ Calcium carbonate is characterized by elevated biocompatibility, non-toxicity, excellent biodegradability, and scalable synthetic availability. This makes it one of the most used materials that has been extensively applied in scientific, environmental, and applicative fields.^10–12^ Recently, CaCO_3_ microparticles, especially spherical ones, have been exploited in different fields as propitious conjugation moieties for a diversity of biological and chemical molecules because of its high specific surface area and high stability.^13,14^ Controlling the surface characteristics of microparticles is crucial in many types of biological applications since they impact the interactions between particles and different biological agents and, thus, the overall performance of the system.^15^

Surface modification of the inorganic microspheres with functional polymer particles (polymer coating) offers a powerful approach to optimize and control the surface features of the microspheres. It increases their chemical affinity through van der Waals force, dipole–dipole interaction, and hydrogen bonding. The functional groups of polymers promote the stable covalent binding of the modified microspheres compound onto the surface of bacteria.^15,16^ The existence of a functional polymeric layer between the microparticle and a biomolecule can improve colloidal stability, reduce nonspecific interaction, and confer peculiar properties to the particles. Through the polymeric coatings, it is also possible to introduce reactive moieties that allow to immobilize biological probes on the particle surface.^17^

Polyvinylpyrrolidone (PVP) is attracting a great deal of interest as a multifunctional material with a wide range of high-performance applications. It is biocompatible, easily processed, and non-antigenic. It was approved by the US Food and Drug Administration as a safe polymer for biological experiments.^18^ It is frequently used due to its interesting properties such as higher glass transition temperature, water solubility, biodegradability, chemical stability. It is also a very good adhesive, and emulsifying agent. It is considered as a good capping agent and because of its superior wetting properties, PVP easily produces homogeneous and resistant coating layers.^19^ PVP can be easily modified using ionizing radiation which is one of the most attractive technologies for modifying polymeric coating. The high energy processed polymers are gaining popularity for wide areas of high-performance application due to several advantages after irradiation of polymers.^19^ Ionizing radiation processing is more convenient, clean, toxic free and environment friendly, it is the most reliable alternative to conventional surface modification approaches^20^

*Bacillus* sp. is known for its versatile use in environmental, medical, and industrial applications. It can produce an array of metabolites, one of which is polyhydroxy butyrate (PHB). PHB is a biopolymer naturally produced by different *Bacillus* sp. and can be induced using optimization of growth culture conditions. It can be used as the base of many biocomposites for its low cost and biocompatibility.^21^ PHB also acts as an energy storage material in bacteria, this increases its viability which helps in sustaining the type of application required.^22^ Therefore, from this standpoint, the aim of the present work is to prepare PHB induced *Bacillus* coated microspheres with CaCO_3_ as the microcarrier and to study the interaction of methylene blue dye with the prepared surface modified biomicrospheres.

## Materials and methods

### Bacteria and cultivation conditions

The bacteria used in the current study was previously isolated, identified as *Bacillus* sp. and characterized and used for nitrate containing textile wastewater.^23^ A single colony was used to inoculate Lauria Bertani media containing 1% NaNO_3_ w/v (99%). The culture was incubated for 24 h at 30 °C at 150 rpm.

### Induction and qualitative determination of PHB production by *Bacillus* sp.

PHB production was qualitatively assayed using Sudan black B dye. with *Bacillus* sp. was streaked on Luria Bertani agar plate and incubated for 24 h. at 37 °C. About 2 mL of ethanolic solution of Sudan black B (0.05% w/v) was added to the plate after colonies were seen on the plates. The plates were incubated for 30 min at room temperature and were later washed with 60% ethanol to remove the excess Sudan Black B from the colonies. The appearance of bluish colonies was recorded as positive for PHB production.^24^

### Optimization of carbon sources for PHB production

*Bacillus* sp. was grown under various carbon sources (glucose, fructose, and sodium pyruvate) for the optimum production of PHB. Briefly, the isolate (1 × 10^8^ cfu per mL) was inoculated in 250 mL Erlenmeyer flask with 50 mL working volume of LB medium supplemented with individual carbon sources (at a final concentration of 1%) at pH 7 and incubated at 37 °C for 48 h.^25^ The produced PHB was extracted and evaluated gravimetrically and by FTIR analysis.

### Effect of inoculum size on PHB production

Fifty mL working volume of LB medium in 250 mL Erlenmeyer flask was adjusted with an initial pH 7. A total of 0.5, 1, 2 and 4% (v/v) seed inoculum (1 × 10^8^ cfu per mL) were transferred into each flask. The optimal incubation temperature was maintained at 37 °C. After 48 h, the culture was centrifuged, and the cells were collected for PHB yield assay.^26^

### Extraction of PHB

The extraction of PHB from *Bacillus* sp. was attained by following the protocol of ref. 24. Fifty mL of PHB production medium with cell biomass was collected and spun in a cooling centrifuge at 10 000 rpm for 10 min and the pellet was washed with an equal volume of acetone and ethanol to rupture the cell wall and remove the unwanted cell debris materials. The supernatant was discarded after the centrifugation of the isolate at 8000 rpm for 5 min and later, the pellet was dissolved in 4% of sodium hypochlorite (NaOCI) and kept at room temperature for 30 min. After 30 min, the contents were spun in a centrifuge at 10 000 rpm for 5 min and the supernatant was discarded. The acquired pellet was splashed with an equal quantity of acetone and ethanol (2 : 1). Polymeric pellet granules were mixed with chloroform and filtered by Whatman No. 1 filter paper (Cat No. 1001090). The percentage yield of PHB was determined using the following equation:
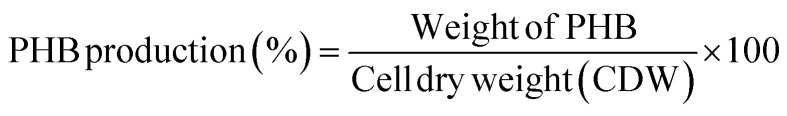


### Measurement of cell dry weight (CDW)

Fifty mL culture broth was centrifuged at 10 000 rpm for 10 min at 4 °C. The supernatant was discarded, and the cell pellets were washed with distilled water. The washed pellet was resuspended in 1 mL of distilled water, transferred to pre weighed aluminum boat and dried to constant weight at 55 °C.^27^

### Factorial design for optimization of PHB production by *Bacillus* sp.

The following factors were used to obtain optimized production and growth of PHB. The Design of Experiment was performed using Minitab® 21.2. Using Full Factorial Design (8 runs). The results were obtained as Pareto Charts, interaction plots and Analysis of Variance with *α* = 0.05. Table 1 shows the chosen factors to be glucose (20, 40 g L^−1^) and pH (7 and 9) and incubation time (2 and 4 days). The response was PHB production (%).

**Table 1:** Experimental design for PHB production by *Bacillus* sp. The factors tested were pH (7 and 9), time (2 and 4 days) and glucose concentration (20 and 40 g L^−1^). (*X*1:−1 and *X*2:+1)

Run	pH	Glucose	Incubation time
1	7	40	2
2	7	40	4
3	7	20	2
4	7	20	4
5	9	40	2
6	9	40	4
7	9	20	2
8	9	20	4

### Synthesis of calcium carbonate microspheres as microcarrier

Calcium carbonate microspheres were synthesized using fast precipitation method.^28,29^ In a typical synthesis, aqueous calcium chloride (0.33 M, purity: 93%) and sodium bicarbonate (0.33 M, purity: 99.8%) precursors were prepared separately beforehand. The precipitation reaction was conducted by addition of the above Na_2_CO_3_ solution into equal volumes of CaCl_2_ aqueous solutions using microdropper. The precipitation of CaCO_3_ was carried out at ambient temperature under continuous stirring of 300 rpm for 1 h. The resulting CaCO_3_ microspheres were then centrifugally separated from the suspension then ultrasonically rinsed with water three times. Finally, the synthesized microspheres were dried in an oven at 60 °C for 12 h.

### Functionalization of CaCO_3_ microspheres

The CaCO_3_ microspheres (1 g) were suspended in a PVP solution (20 mL, purity: 99%) of concentration 1%. The suspension was gently stirred at room temperature for 24 h. The suspension was then poured into glass bottles and then irradiated at 5 kGy.^19^ The irradiation process was performed at dose rate 0.99 kGy h^−1^ at room temperature in a ^60^Co gamma irradiation facility located at National Center for Radiation Research and Technology (NCRRT), Egyptian Atomic Energy Authority, Cairo, Egypt. Afterwards, the functionalized microspheres were centrifuged, copiously washed with water several times and then dried at 50 °C for 24 h to obtain the functionalized microspheres (CaCO_3_/PVP).

### Preparation of *Bacillus* sp. coated calcium carbonate microspheres

*Bacillus* sp. with highest PHB production was used to coat calcium carbonate microspheres. About 1 : 1 ratio of *Bacillus* sp. to surface modified calcium carbonate microspheres and incubated for 24 h at 30 °C. The overnight composite was centrifuged and washed with sterile distilled water. The precipitate was dried overnight at 50 °C and used in the bioremoval experiment.

### Structural and surface characterization of the prepared and functionalized CaCO_3_ microspheres

The FT-IR spectra were acquired from 4000 to 400 cm^−1^ at a resolution of 4 cm^−1^, using a Bruker Vertex 70 FT-IR spectrometer, Germany. XRD analyses were performed on a Shimadzu machine (XRD-6000 series) with Cu-Kα radiation (*λ* = 1.54 A), operated at 40 kV and 30 mA. XRD patterns were recorded in the range of 2*θ* = 4–90° (by steps of 0.02°). Scanning Electron Microscopy (SEM) was used to capture the images of the prepared microspheres on each stage. Images were captured using Zeiss evo 15 scanning electron microscope (Germany). Microspheres were placed on brass stubs using double-sided adhesive tape and coated with a thin layer of gold under reduced pressure. The images were captured at magnifications of 400 and 5000× using an electron beam high voltage of 20 kV.

The mean particle sizes and zeta potentials of the prepared biocarriers were determined by Dynamic light scattering (DLS) using a PSSNICOMP particle sizer 380ZLS (PSS-NICOMP, Santa Barbara, CA, USA). The zeta potential measurements were performed by applying an electric field of strength 10 V cm^−1^ using three 30 s cycles. The results were outlined as a mean result of at least five readings on triplicate samples.

### Bioremoval of methylene blue

Surface modified calcium carbonate microspheres, *Bacillus* sp, coated calcium carbonate microspheres, extracted PHB and mixture of PHB and biomicrospheres were used to remove methylene blue. About 100 mg of each were incubated with 25, 50 and 100 mg per L methylene blue. The mixture was incubated at 30 °C under static conditions. A UV-Visible scan was performed for each sample and control (methylene blue dye). The scan was performed from 400–800 nm using spectrophotometer (SPECORD 210 plus, analytic Jena). The time and location tests were performed at 0, 1 and 18 h incubation using 0, 25, 50 and 100 mg of microspheres in cell culture plates and the images were captured using a digital camera. Decolorization was calculated according to the following equation:
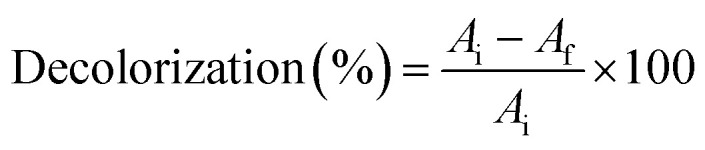
where *A*_i_ is the initial dye absorbance and *A*_f_ is the final dye absorbance. Standard curve for MB was prepared by using different MB concentrations of 12.5, 25, 50 and 100 mg L^−1^, the absorbance of each concentration was plotted against the concentration and linear fit equation was used to calculate residual MB concentrations.

## Results

### PHB confirmation test

**Fig. 1:**
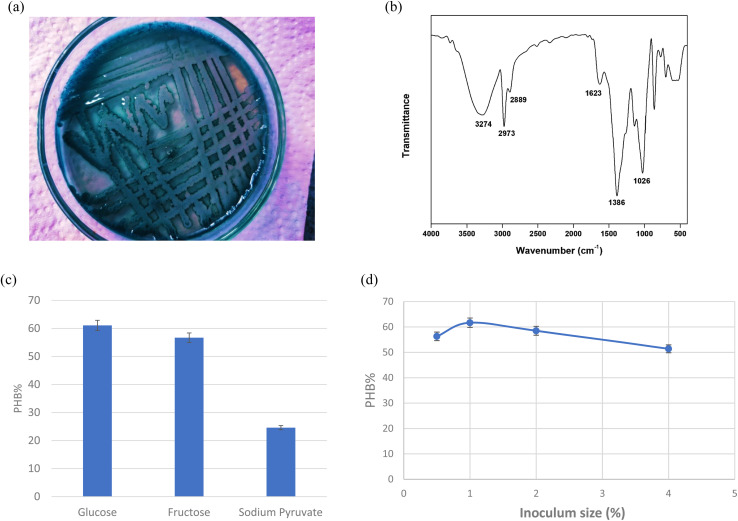
(a) Blue colonies of *Bacillus* sp. stained with Sudan black to indicate the production of PHB. (b) FTIR spectrum for *Bacillus* sp. PHB. (c) Effect of adding different carbon sources on *Bacillus* sp. PHB production%. (d) Effect of adding different inoculum size on *Bacillus* sp. PHB production%.

The Sudan Black B stained isolate showed dark greenish blue colored colonies on LB agar plates (Fig. 1a). The produced PHB was extracted, dried and the powder was used for FTIR spectroscopy analysis. FTIR results (Fig. 1b) shows peaks at 3274 cm^−1^ characteristic for O–H (stretching mode) carboxylic acid, 2973 cm^−1^ and 2889 cm^−1^ are characteristic for CH_3_, CH_2_, 1623 cm^−1^ characteristic for C=O conjugation of aldehyde with aromatic ring, 1386 cm^−1^ characteristic for C–O–H bending mode and 1026 cm^−1^ characteristic for C–O alcohol. The results in Fig. 1c shows that the highest PHB production was 61.09%, it was obtained upon adding glucose to the media, while PHB reached 56.7 and 24.6% when adding fructose and sodium pyruvate to the media, respectively. Fig. 1d represents the effect of inoculum size on the production of PHB. The results show that the highest production was obtained when using 1% inoculum size which reached 61.67. While the lowest value was 51.4% when 4% inoculum size was added to the media.

### Factorial design

**Fig. 2:**
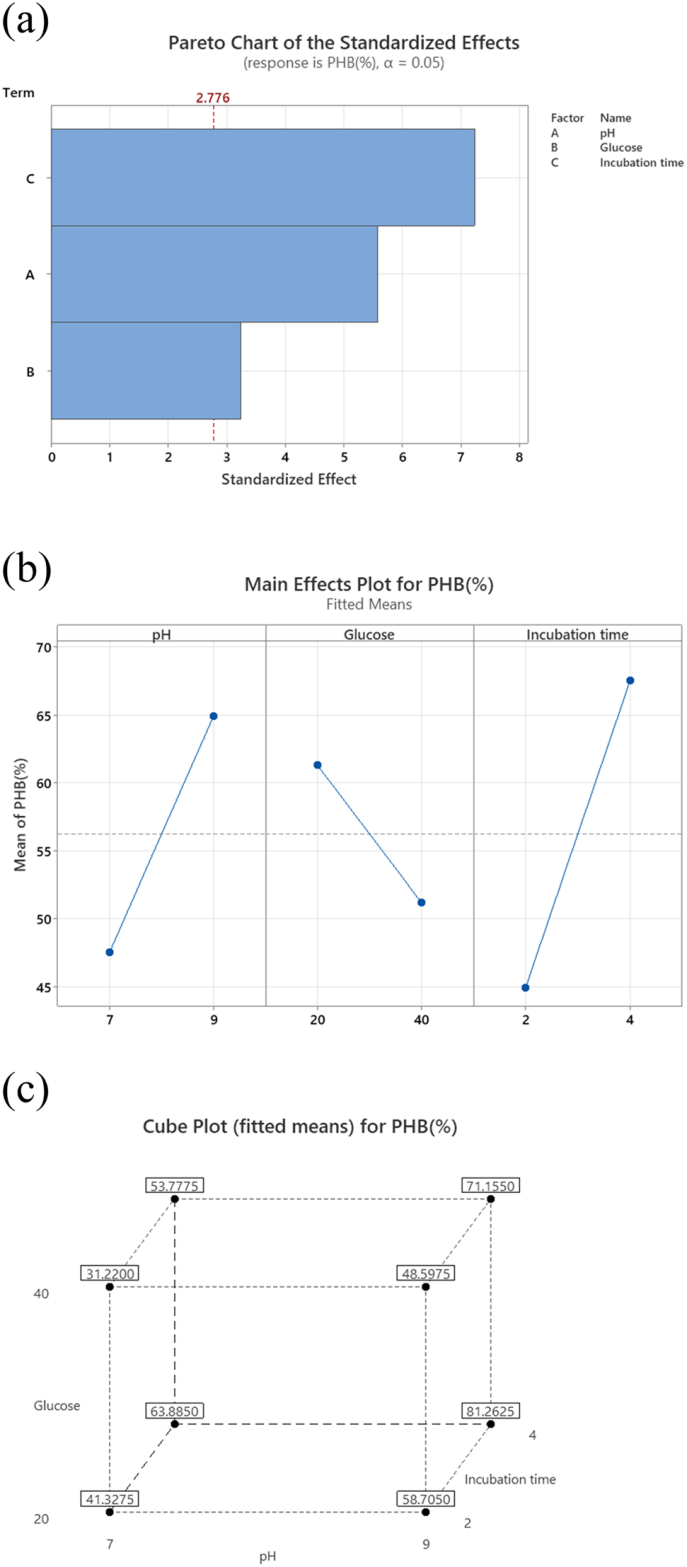
(a) Pareto chart for PHB production (%) for *Bacillus* sp. Factorial design. (b) Main effects plot for PHB production (%) for *Bacillus* sp. Factorial design. (c) Pareto chart for PHB production (%) for *Bacillus* sp. Factorial design.

The results shown represent the results after performing full factorial design. Pareto chart (Fig. 2a) indicates that the order of significance of the testes factors were pH > incubation time > glucose while main effect result (Fig. 2b) shows that the optimal conditions for PHB production by *Bacillus* sp. is at pH 9, 20 g per L glucose concentration and 4 days of incubation.

Cube plot (Fig. 2c) shows that the highest PHB% production was 81.26 for cultures prepared at initial pH 9, 20 g per L glucose and incubated for 4 days, this is followed by PHB% production was 71.50 for cultures prepared at initial pH 9, 40 g per L glucose and incubated for 4 days which indicates that the highest values are reached at pH 9 and incubation time of 4 days as primary effective factors. The value of PHB% decreases at pH 7 and 2 days incubation time to reach 31.2. The model summary, analysis of variance and regression equation are shown in Table 2. The table shows that *P*-value for pH and incubation time were 0.005 and 0.002, respectively, while that for glucose was 0.032. The *R*-Sq value was 95.92%, *R*-Sq (adjusted) was 92.86% and that for *R*-Sq (predicted) was 83.67%.

**Table 2:** Model summary, analysis of variance and regression equation for factorial design

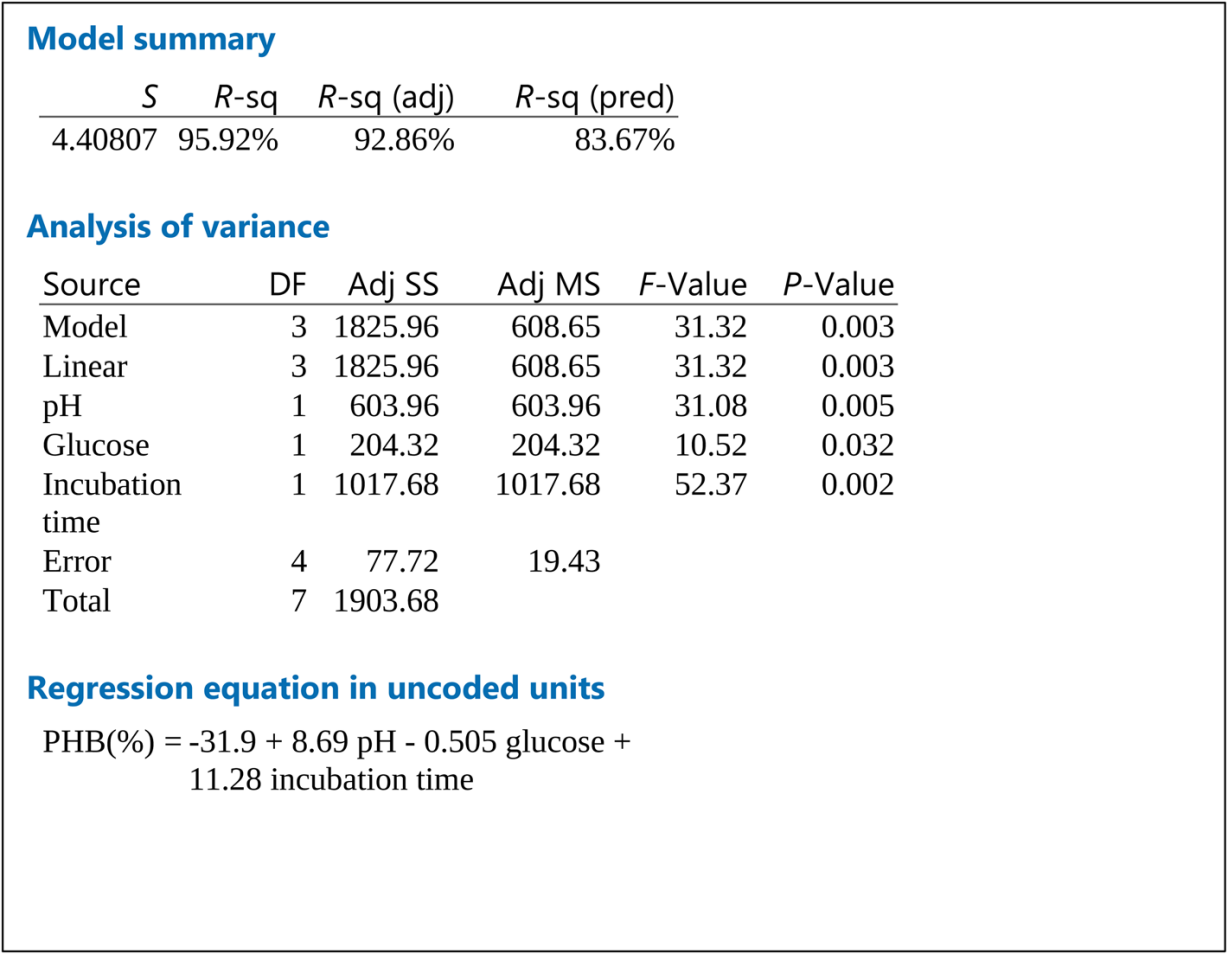

### Calcium carbonate microspheres preparation and surface modification

**Fig. 3:**
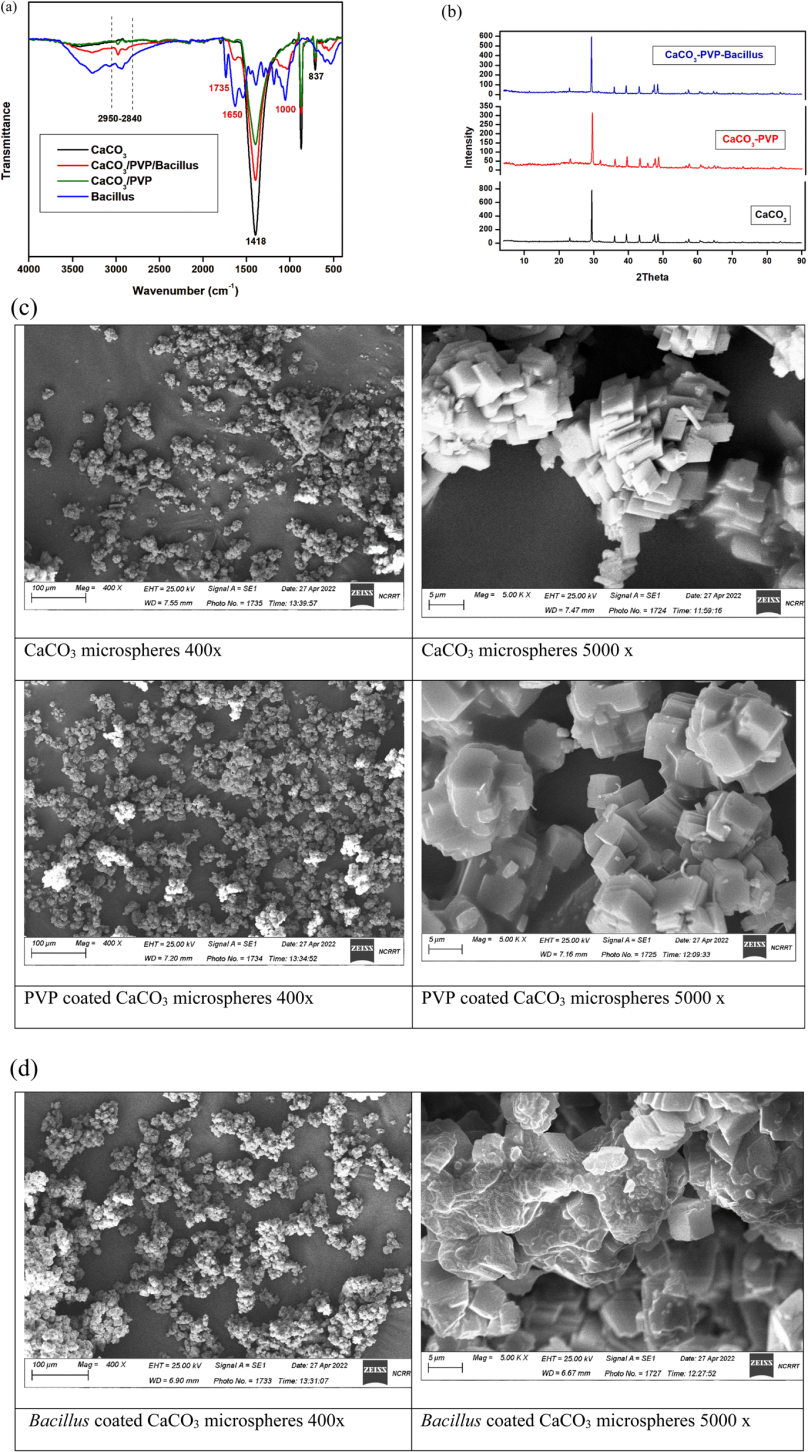
(a) FTIR for different microsphere preparations. (b) XRD for different microsphere preparations. (c) SEM images representing the preparation steps of *Bacillus* coated CaCO_3_ microspheres at magnifications 400 and 5000×.

Preparation of surface modified calcium carbonate microspheres was confirmed at each step using FTIR, XRD, DLS, zeta potential and SEM images. From the FTIR spectrum of CaCO_3_ nanoparticles illustrated in Fig. 3a. The spectrum shows that the nano CaCO_3_ had adsorption bands at 2950–2840 cm^−1^, corresponding to the vibration mode of C–H of stearic acid, and also 707, 873 and 1418 cm^−1^ corresponding to the in-, out-plane bending and asymmetrical stretching vibration peaks of O–C–O, respectively. In the CaCO_3_/PVP, the characteristic bands of PVP at 2950 and 1652 cm^−1^ proved the existence of asymmetric stretching of CH_2_ and stretching of C–O, respectively. The spectrum for *Bacillus* sp. coating the CaCO_3_/PVP microspheres shows an extra peak at 1735 cm^−1^ that corresponds to ester carbonyl bond and those between 1650 and 1000 cm^−1^ corresponding to proteins in carbohydrates.

The phase of the synthesized CaCO_3_ microspheres was identified by XRD characterization. As shown in Fig. 3b, every diffraction peak is in agreement with the standard diffraction patterns of calcite (JCPDS 24-0027), the characteristic peaks at 2*θ* of 22.87°, 29.14°, 35.70°, 39.14°, 42.97°, 47.28°, 48.25°, 56.45° and 57.27° are corresponding to (012), (104), (006), (113), (202), (024), (116), (211) and (122) crystallographic nucleation planes of calcite phase, respectively. Among these planes, high intensity is observed for 104 plane (29.14°) which is the characteristic plane of rhombohedral CaCO_3_. SEM images represent the preparation steps (Fig. 3c) where the *Bacillus* cells can be seen coating CaCO_3_/PVP microspheres. The size and charge of each preparation is represented in Table 3. Zeta potential represents the charge on the modified microspheres. The charge increases from −7 and −8 mV for CaCO_3_ and CaCO_3_/PVP, respectively to −18 and −16 mV for *Bacillus* coated microspheres. The size increased from 1.617 μm in CaCO_3_ microspheres to 1.892 μm after coating with PVP and 2.34 μm after coating the final layer of PHB induced *Bacillus* sp.

**Table 3:** Size and net charge of different prepared microspheres detected by dynamic light scattering and zeta potential

Sample	Size (μm)	Zeta (mV)
CaCO_3_	1.617	−7
CaCO_3_/PVP	1.892	−8
CaCO_3_/PVP/Bacillus	2.315	−18
CaCO_3_/PVP/Bacillus-PHB	2.34	−16

### Use of surface modified calcium carbonate microspheres coated with *Bacillus* sp. in methylene blue dye removal

**Fig. 4:**
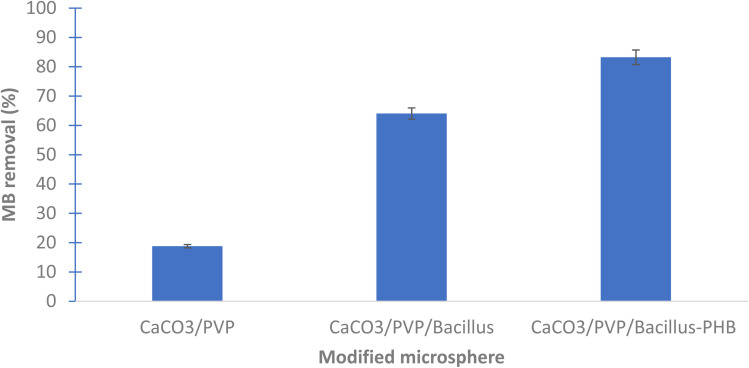
Bioremoval of methylene blue using different microsphere preparations.

**Fig. 5:**
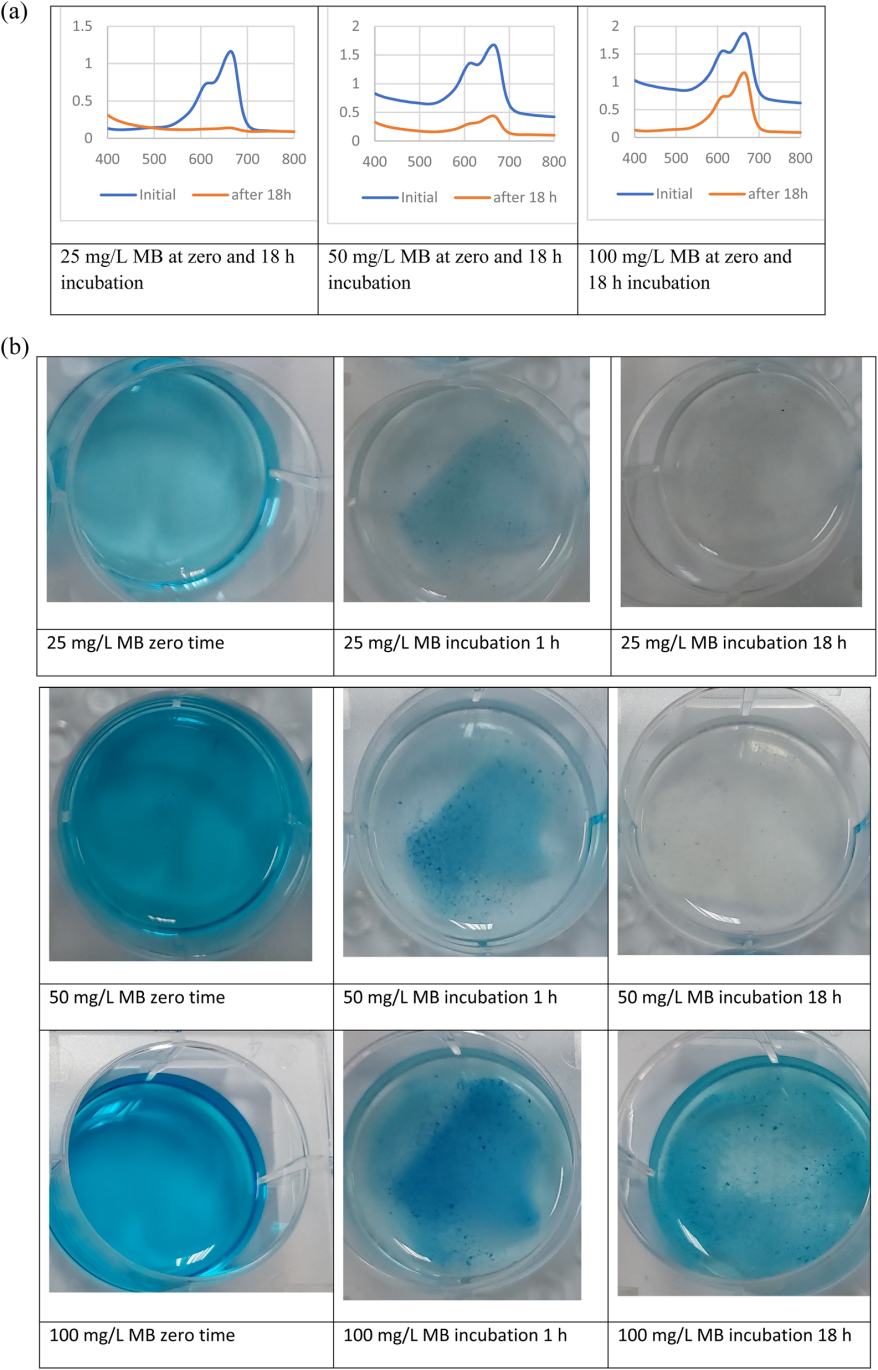
(a) Removal of different methylene blue dye concentrations at different time points using surface modified calcium carbonate microspheres coated with *Bacillus* sp. at 30 °C and static conditions. (b) Photos of time dependent removal of different methylene blue dye using surface modified calcium carbonate microspheres coated with *Bacillus* sp. at 30 °C and static conditions.

**Fig. 6:**
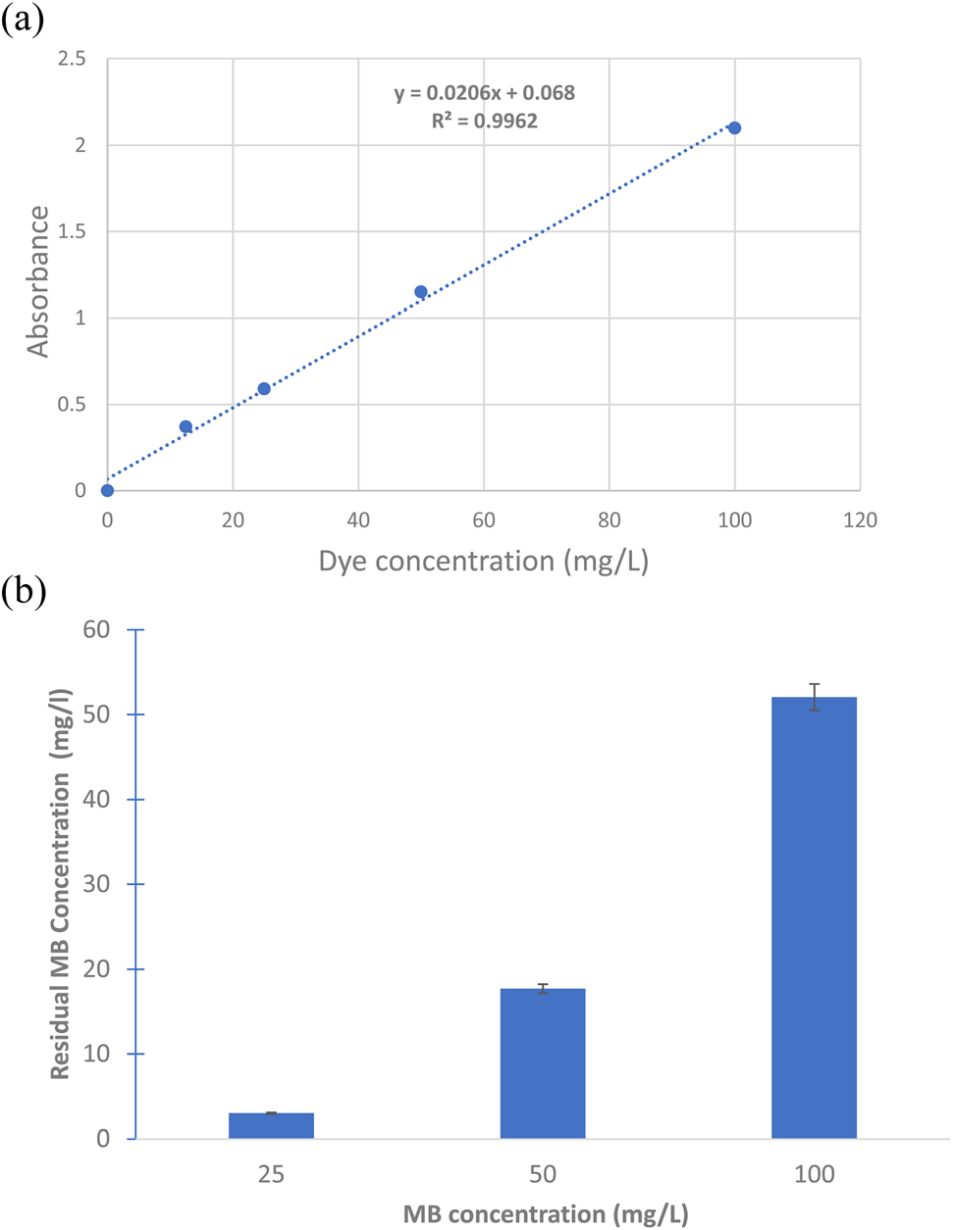
(a) Standard curve for methylene blue dye using different dye concentrations displaying equation and R sq value on chart. (b) Residual methylene blue dye concentration after incubation for 18 h with surface modified calcium carbonate microspheres coated with *Bacillus* sp. at 30 °C and static conditions. Calculations obtained from standard curve equation in (a).

The results show that the highest removal of MB dye was when *Bacillus* sp. cells were used in the coating of the prepared microspheres where the decolorization reached 64.05 and 88.23% for CaCO_3_/PVP/*Bacillus* and CaCO_3_/PVP/PHB induced *Bacillus*, respectively, while that for CaCO_3_/PVP was 18.79% (Fig. 4). When CaCO_3_/PVP/PHB induced *Bacillus* were added to different MB concentrations (25, 50 and 100 mg L^−1^) using 100 mg microspheres and the decolorization was monitored at different time intervals. Fig. 5a represents UV-visible spectrum at 18 h incubation. The spectra show a decrease in peaks for all tested concentrations. Fig. 5b represents the images for the time-based decolorization. The images show initial adsorption of MB to the microsphere after 1 h incubation. MB decolorization took place after 18 h for 25 and 50 mg per L MB concentrations, while 100 mg per L MB showed distinct blue color of MB on the microspheres. Calculating the residual dye concentration was obtained using the equation on standard curve chart (Fig. 6a). The residual dye concentrations were calculated to be 3, 17.7 and 52.04% for initial dye concentrations of 25, 50 and 100 mg L^−1^, respectively (Fig. 6b). Outline of the preparation process and decolorization pathway is found in graphical representation shown in Fig. S1.†

**Fig. 7:**
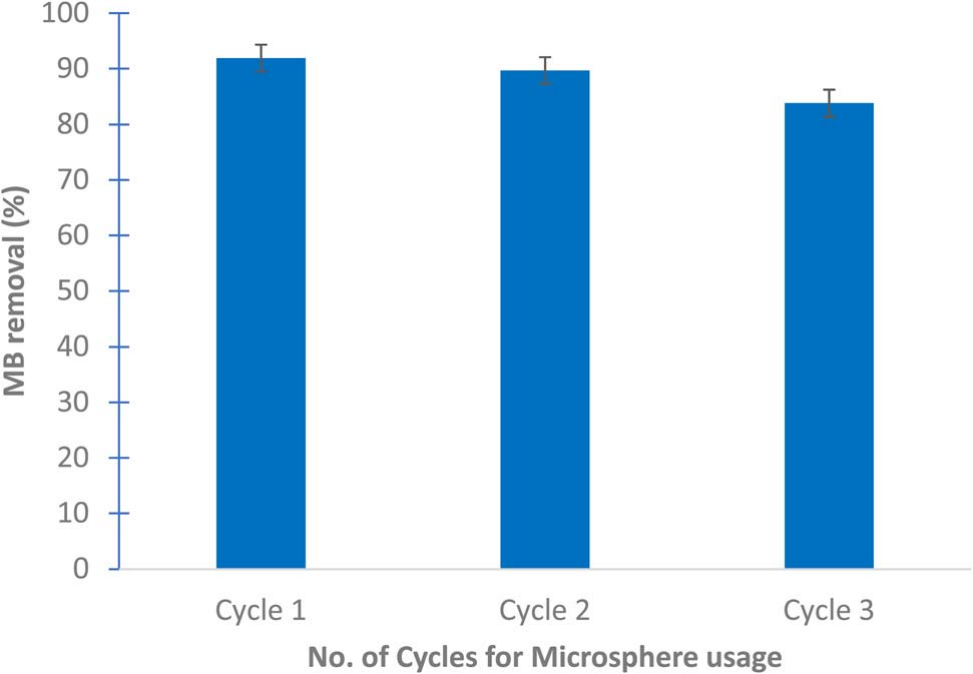
MB removal over 3 cycles using 25 mg microspheres and 25 mg per L MB dye after 18 h at 30 °C and static conditions. No dye desorption was performed.

To test the multiple usage of the CaCO_3_/PVP/PHB induced *Bacillus* microspheres, they were used 3 times without desorption. Fig. 7 shows that MB decolorization was not affected by the multiple usage. The results show 91.89, 89.65 and 88.78% decolorization for cycles 1, 2 and 3, respectively.

## Discussion

*Bacillus* sp. under study was previously reported to decolorize dyes, contribute to electron transfer, and to produce different metabolites such as butyrate suggesting the production of PHB. In addition to that, it was able to form biofilm on activated carbon.^23^ The present work highlights both attributes by focusing more on the use of *Bacillus* sp. as coating on calcium carbonate microspheres and at the same time inducing PHB production to increase tolerance to xenobiotics and provide energy storage material to enhance the longevity and performance of the prepared *Bacillus* coated microspheres. The blue color of *Bacillus* sp. colonies stained with Sudan Black B indicates that the bacteria can accumulate PHB. Sudan Black B is a good indicator for presence of PHB since lipophilic granules adsorb Sudan Black B stain due to its high affinity with lipid-based molecules that turns the colonies bluish.^30^

The FTIR for the produced PHB shows similar peaks to those reported in literature.^31^ For maximum PHB production, it is important to assess the physiological and environmental factors of the bacterial isolate under examination for the PHB production process. Factorial design is an efficient way to screen the nutritional and physical factors among several process variables, affecting Poly-β-hydroxybutyrate production by selected *Bacillus* sp. Our results are in accordance to ref. 32 who reported similar PHB content. Ref. 33 showed that the highest PHB content% was obtained at pH7 and 9, respectively. Ref. 34 also suggested that alkaline conditions could improve the yield of PHB. Higher environmental pH-values indeed might trigger intracellular concentrations of organic acids.^35^ These acids can in turn be converted into PHA, which is further stimulated by the excess amount of carbon in the cultures. This hypothesis might explain why more PHB is accumulated under alkaline conditions. During PHB production at pH 7 and 9, substrates are used to grow cells and accumulate energy in the initial stage of PHB accumulation. Incubation time is an important factor associated with bacterial growth and PHB accumulation. The results revealed that after 4 days incubation in the medium under the stationary phase of growth, the highest PHB production and cell dry weight were 1 and 1.350 g L^−1^ (74.04% PHB content), respectively. These results are consistent with ref. 36 and 37 who stated that PHB concentration at 1 day incubation was minimal because the culture was in the acclimatization period, and reached the highest production at 4 days, above which the production decreases. This may be due to a lack of nutrients and an increase in metabolites, toxins and inhibitors, that might have a negative effect on PHB synthesis. The third factor tested for optimal PHB production was glucose. The glucose concentrations were selected based on the results achieved by ref. 37 who found that the higher PHB production was observed at glucose concentration 20 g L^−1^. On the other hand, ref. 38 showed that higher growth and maximum PHA production was detected in the glucose medium 40 g L^−1^. Our results show that glucose contribution was the least of the three tested factors as seen from the factorial design.

After growing PHB induced Bacillus cells, it was added to surface modified calcium carbonate microspheres and confirmation of each step was performed using different approaches. FTIR spectrum confirms the presence of characteristic peaks in calcite. No obvious differences were observed between the spectra of CaCO_3_/PVP and CaCO_3_ indicating that no reaction between CaCO_3_ and PVP was apparent. By comparing the CaCO_3_/PVP/*Bacillus* spectrum with *Bacillus* and CaCO_3_/PVP spectra, overall, the peaks of both bacteria and carbonate calcium are present in the CaCO_3_/PVP/*Bacillus*, but at different intensity levels and with the appearance of carbonyl group and glycoproteins.^23^ These results confirm that each layer perfectly makes a coating on CaCO_3_ microspheres without any chemical interaction. XRD shows that the polymer coating led to a diminished intensity for 29.14° peak and a change in the degree of crystallization combined with increases in the amorphous regions. This increase in the amorphous regions showed that the PVP coating was successfully constructed on the surface CaCO_3_ microspheres. SEM results also show that bacteria were attached to the microspheres. The shape and size of the finally prepared microspheres is in accordance to ref. 9. Zeta potentials of all microspheres were determined to follow the change in the surface potential after each coat. The results indicate that the surface charge of calcium carbonate decreases to more negative values with addition of *Bacillus*. The change in surface potential of calcium carbonate is due to adsorption of PVP and *Bacillus* on the surface of the CaCO_3_ microspheres. *Bacillus* was reported to possess a net negative charge, and this is the cause for the increased negativity of the surface modified microsphere. Gram positive bacteria has a net negative charge due to the presence of teichoic acid in their cell wall.^39^ The size of the prepared CaCO_3_ microspheres and its surface modifications are in accordance to ref. 9 who reported the average size to be 3–5 μm.

Although MB dye decolorization was previously reported to take place efficiently *via* adsorption using natural or synthesized material,^5–8^ yet our work presents a time and location dependent removal of MB that can be tailored based on the intended removal purpose, weather its complete decolorization, recovery of MB dye. Spatiotemporal study allows for diverse applications. PHB induction is reported to alleviate heat stress in bacteria.^40^ It is expected that its presence would help *Bacillus* sp. under study as a protectant to hostile environmental conditions. Ref. 9 used multiple steps for desorption of dyes prior to re-use, our work demonstrates multiple usage of the prepared microspheres without desorption steps which makes it more practical. Moreover, the MB was adsorbed onto the microspheres as indicated by the decrease in absorption intensity of the 2 distinctive peaks of MB, this is contrary to our results which show that time dependent adsorption can be changed to complete decolorization as indicated by the disappearance of both MB peaks after 18 h of incubation. It is noteworthy to mention that ref. 9 reported 76.61% as its highest decolorization after 18 days. The change in adsorption and decolorization for the surface modified microspheres can be seen clearly with the naked eye. CaCO_3_ microspheres are characterized by their large surface area.^41^ The core–shell preparations such as that for CaCO_3_ microspheres.^9^ Core-shell 2-layer model form layers that can be tightly or loosely bound, this helps in the removal of adsorbates.^42^ This model provides a way for MB to seep inside the microspheres, providing more surface area for MB adsorption and decolorization.

The prepared CaCO_3_/PVP/PHB induced *Bacillus* microspheres have the advantage of using live bacterial cells with reductase enzyme system that is located on the outer cell membrane, it is composed of Mo cofactor, Fe–S cluster and a heme containing cytochrome connected to a quinone pool.^23^ This makes the decolorization of MB a biological process that can be repeated several times. In the present study, the surface modified microspheres were used 3 times with the same efficiency without the need for desorption. This is contrary to CaCO_3_ microspheres that needed desorption at 450 °C for 2 h prior to its re-use.^9^

## Conclusion

The morphology and characteristics of the prepared surface modified CaCO_3_ microspheres coated with PHB induced *Bacillus* sp. can direct its usability and choice of application. An initial adsorption of dyes can take place due to the surface charge of *Bacillus* sp. biofilm while after longer incubation, decolorization takes place *via* the multiplex of enzymes and metabolites. The prepared microspheres can be used multiple times without the need for desorption since the intrinsic enzyme system of *Bacillus* performs the decolorization efficiently over the course of the 3 cycles. The prepared microsphere can also be used for drug delivery since all its components are safe to use *in vivo*. This is the focus of our future work.

## Ethical statement

This article doesn't contain any studies with participation or animals performed by any of the authors.

## Author contribution

N. D. conceived and designed research, conducted experiments, wrote manuscript, H. B. conceived and designed research, conducted experiments, wrote manuscript, O. G. conceived and designed research, conducted experiments, wrote manuscript and was responsible for final editing. All authors read and approved the manuscript.

## Conflicts of interest

Author 1 declares that she has no conflict of interest, author 2 declares that she has no conflict of interest, author 3 declares that she has no conflict of interest.

## Supplementary Material
